# Long-term voluntary wheel running does not alter vascular amyloid burden but reduces neuroinflammation in the Tg-SwDI mouse model of cerebral amyloid angiopathy

**DOI:** 10.1186/s12974-019-1534-0

**Published:** 2019-07-11

**Authors:** Lisa S. Robison, Dominique L. Popescu, Maria E. Anderson, Nikita Francis, Joshua Hatfield, Joseph K. Sullivan, Steven I. Beigelman, Feng Xu, Brenda J. Anderson, William E. Van Nostrand, John K. Robinson

**Affiliations:** 1 Department of Psychology, Stony Brook University, 100 Nicolls Road, Stony Brook, NY 11794 USA; 2 Present Address: Department of Neuroscience and Experimental Therapeutics, Albany Medical College, 47 New Scotland Ave, Albany, NY 12208 USA; 3 Present Address: George and Anne Ryan Institute for Neuroscience and Department of Psychology, University of Rhode Island, 130 Flagg Road, Kingston, RI 02881 USA; 4 Present Address: Department of Psychology, Farmingdale State University, 2350 Broadhollow Rd, Farmingdale, NY 11735 USA; 5 George & Anne Ryan Institute for Neuroscience and Department of Biomedical and Pharmaceutical Sciences, University of Rhode Island, 130 Flagg Road, Kingston, RI 02881 USA; 6 Present Address: New York Medical College, School of Medicine, 40 Sunshine Cottage Rd, Valhalla, NY 10595 USA

**Keywords:** Cerebral amyloid angiopathy, Alzheimer’s disease, Exercise, Cardiovascular, Aerobic, Fitness, Beta amyloid, Inflammation, Anti-inflammatory

## Abstract

**Background:**

Cardiovascular exercise (CVE) has been shown to be protective against cognitive decline in aging and the risk for dementias, including Alzheimer’s Disease (AD). CVE has also been shown to have several beneficial effects on brain pathology and behavioral impairments in mouse models of AD; however, no studies have specifically examined the effects of CVE on cerebral amyloid angiopathy (CAA), which is the accumulation of amyloid-beta (Aβ) in the cerebral vasculature. CAA may be uniquely susceptible to beneficial effects of CVE interventions due to the location and nature of the pathology. Alternatively, CVE may exacerbate CAA pathology, due to added stress on already compromised cerebral vasculature.

**Methods:**

In the current study, we examined the effects of CVE over many months in mice, thereby modeling a lifelong commitment to CVE in humans. We assessed this voluntary CVE in Tg-SwDI mice, a transgenic mouse model of CAA that exhibits behavioral deficits, fibrillar vascular Aβ pathology, and significant perivascular neuroinflammation. Various “doses” of exercise intervention (0 h (“Sedentary”), 1 h, 3 h, 12 h access to running wheel) were assessed from ~ 4 to 12 months of age for effects on physiology, behavior/cognitive performance, and pathology.

**Results:**

The 12 h group performed the greatest volume of exercise, whereas the 1 h and 3 h groups showed high levels of exercise intensity, as defined by more frequent and longer duration running bouts. Tg-SwDI mice exhibited significant cerebral vascular Aβ pathology and increased expression of pro-inflammatory cytokines as compared to WT controls. Tg-SwDI mice did not show motor dysfunction or altered levels of anxiety or sociability compared to WT controls, though Tg-SwDI animals did appear to exhibit a reduced tendency to explore novel environments. At all running levels, CAA pathology in Tg-SwDI mice was not significantly altered, but 12-h high-volume exercise showed increased insoluble Aβ burden. However, CVE attenuated the expression of pro-inflammatory cytokines TNF-α and IL-6 and was generally effective at enhancing motor function and reducing anxiety-like behavior in Tg-SwDI mice, though alterations in learning and memory tasks were varied.

**Conclusions:**

Taken together, these results suggest that CAA can still develop regardless of a lifespan of substantial CVE, although downstream effects on neuroinflammation may be reduced and functional outcomes improved.

## Background

Due to the lack of broadly effective pharmaceutical treatments, discovering means of preventing and/or treating Alzheimer’s disease (AD) and related dementias is a high priority. There has been growing interest in research to identify modifiable lifestyle factors that may reduce the risk and/or affect the progression of AD. Epidemiological studies have identified regular physical activity as a protective factor against cognitive decline and AD [1, 2]. Greater physical activity levels are linked to enhanced cognitive performance in healthy older adults, suggesting that the effects of exercise may be dose-dependent [3]. Results from longitudinal studies are also positive, linking mid-life physical activity to reduced risk of cognitive decline and dementia in later years [4, 5]. In a randomized controlled trial, 24 months of moderate intensity exercise had no effect on cognitive performance in previously sedentary older adults [6], suggesting that earlier, longer, or even life-long exercise interventions may be necessary to be effective. These findings in healthy populations have led researchers to assess whether exercise may also be therapeutic for patients with AD and other dementias; however, randomized controlled trials investigating the efficacy of exercise for reducing AD are severely lacking. A randomized controlled trial of 200 patients with mild AD found that a 16-week moderate- to high-intensity aerobic exercise program (60 min, three times per week) resulted in attenuated decline on the Neuropsychiatric Inventory [7]. Additionally, adherence to treatment was associated with improved performance on the Symbol Digit Modalities Test, which assesses mental speed and attention [7].

Benefits of exercise in murine models of AD have also been observed for both neuropathology and cognitive performance, though positive results have not been universal [8–15]. Most studies have used voluntary wheel running, though daily access periods, duration of exercise, and age at the start of intervention have varied considerably among studies. Additionally, various murine models of AD have been used that present considerable variation in type and onset of pathology. This lack of consistency raises numerous questions about exercise volume, when intervention must begin and for how long it must be maintained, is necessary to see benefits, and which particular aspects of pathology are most likely to be improved.

Vascular amyloid pathology (cerebral amyloid angiopathy (CAA)) would seem to be a particularly ripe target for exercise intervention. CAA is a neurological condition in which amyloid is deposited in and along the walls of the vasculature in the central nervous system, resulting in a robust neuroinflammatory response and intracerebral microbleeds and hemorrhages. CAA is common, with an estimated 20–40% of individuals in the general population presenting with CAA pathology upon autopsy [16]. CAA is also an important component of AD, as over 80% of individuals with AD also exhibit at least mild CAA pathology [16]. It has been shown that there is a strong positive correlation between CAA and AD symptoms and pathology [17], and CAA appears to be linked to deficits in perceptual speed and episodic memory, even after controlling for age and AD pathology [18]. Therefore, determining the effects of exercise on CAA and associated deficits would be of particular interest, since CAA may be differentially susceptible to this intervention due to the vascular nature of the pathology. One could predict two opposite functional outcomes of exercise, with the first hypothesis being one where exercise is beneficial due to a reduction of vascular-associated pathology, and the other where exercise is detrimental due to placing additional stress on an already compromised cerebral vascular network.

To evaluate these hypotheses, we conducted a study that utilized the Tg-SwDI mouse, a well-characterized mouse model of CAA, and examined the physiological, neuropathological, and behavioral effects of voluntary cardiovascular exercise (CVE). To evaluate *how much* daily exercise was needed to produce effects, we assessed multiple daily “doses” (access periods) of exercise (1 h, 3 h, or 12 h of daily running wheel access) beginning at 3.5–4 months of age in Tg-SwDI mice compared to sedentary WT and Tg-SwDI controls. Additionally, we assessed the efficacy of a long treatment period (8 months), as compared to previous animal studies that utilized exercise intervention periods of 4 months or less, to model the effects of a lifetime of CVE. We compare the dose-dependent effects of lifelong CVE in the Tg-SwDI model of CAA to our previous work utilizing the same exercise regimens in similarly aged C57BL/6 mice, which found that exercise significantly influences several physiological and behavioral measures and that even relatively small amounts of daily exercise can be beneficial [19].

## Methods

### Animals

C57BL/6 WT (*n* = 10) and Tg-SwDI (*n* = 40) mice, split equally between males and females, were used in this experiment. One Tg-SwDI mouse (assigned to the 12 h exercise group) died before behavioral assessment, so this animal’s data was excluded from all analyses. The Tg-SwDI mouse is a model of CAA, in which fibrillar Aβ accumulates in the cerebral vasculature [20], apparently due to increased fibrillogenicity and/or insufficient clearance of the peptide across the blood-brain barrier [21]. These mice are on a C57BL/6 background and express low levels of human amyloid beta-precursor protein (APP) gene containing the Swedish K670N/M671L, Dutch E693Q, and Iowa D694N mutations, under the control of the mouse *Thy1* promoter [20]. CAA pathology is accompanied by vascular degeneration and marked neuroinflammation [22], as well as impaired Barnes maze performance not attributable to deficits in mobility, strength, or coordination [23].

Mice were housed in a controlled room (22 ± 2 °C and 40–60% humidity) with a 12-h reverse light-dark cycle (lights off 0800 h). Mice habituated for 1 week prior to the beginning of the experiment, then split into experimental treatment groups at 3.5–4 months of age. Purina Lab Diet chow was available ad libitum, and body weight and food intake were recorded weekly throughout the entire experiment. All experiments were conducted in conformity with the National Academy of Sciences Guide for Care and Use of Laboratory Animals and approved by the Stony Brook University Institutional Animal Care and Use Committee.

### Voluntary exercise intervention

Exercise intervention began at 3.5–4 months of age, a time point when memory deficits are apparent and CAA pathology has begun to develop [23, 24]. The intervention lasted for 8 months, with mice being euthanized at approximately 12 months of age. Behavioral deficits and pathology for Tg-SwDI mice have been well described at this age point, with low rates of mortality [23, 25]. Throughout the intervention period, mice were given access to a running wheel 5 days per week for 1 h, 3 h, or 12 h per day. Voluntary wheel running was chosen over forced exercise, such as a treadmill running regimen, so that mice could choose when and how much to run. This minimizes potential confounding variables introduced by forced exercise, such as stress [26]. Additionally, direct comparison of voluntary and forced exercise (treadmill running) in a mouse model of AD found that wheel running was superior to treadmill running in rescuing cognitive deficits and reducing pathology [12].

### Running analyses

Running patterns were recorded in detail to characterize patterns of activity throughout sessions, as we have done previously using the same exercise regimens in C57BL/6 mice [19]. The number of wheel rotations performed per minute was recorded. Running behavior was analyzed to determine differences in running volume (total amount of running per day) and “intensity” measures (the maximum speed and clustering of running bouts that produce higher levels of short-term metabolic demand) between exercise groups and determine their effects on behavior and/or pathology. Average rotations per minute (minute bins with > 5 rotations; “speed”) and breaks per hour (number of minute bins with < 5 rotations in each hour) were calculated as measures of running intensity.

### Behavioral assessment battery

All mice underwent a battery of behavioral tests following the 8-month intervention period, including rotarod, open field, social interaction, marble burying, object placement, novel object recognition, Y-maze for spontaneous alternation, and Barnes maze. These procedures are described in detail in [19]. All animals had been exposed to pre-training in the behavioral paradigms prior to onset of the exercise paradigm and at 4 months into training except for Barnes maze, which was only performed once following 8 months of intervention. All behavior, except for rotarod, was recorded and analyzed using ANY-maze™ software.

The rotarod was performed to assess balance, strength, and motor coordination as done previously [19]. Mice were placed on the rotarod (model ENV-575 M; Medical Associates Inc.), with increasing speed of up to 40 revs/min over a five-minute period. The time on rod until the mouse fell (max time = 300 s) was recorded. Mice were tested three times, with a minimum 5-minute inter-trial interval, and the average of the best two trials were used for analysis.

For the open field, mice were placed in a square 60 cm × 60 cm open field arena for 10 min. General locomotor behavior and motor function was assessed using the measure of distance traveled in the open field. Anxiety-like behavior was assessed by measurement of center activity.

Crawley’s three chamber paradigm was adapted to assess sociability [27], as described previously [19]. This test consisted of two 5-min trials that occurred consecutively, with the first trial serving as habituation to the empty apparatus. In the second trial, the middle chamber was empty, one side chamber contained an empty cup, and the other side chamber housed another mouse in an identical cup (Stranger 1). Location of Stranger 1 (left or right side) was randomly assigned. Sociability was assessed by comparing time spent with the cup containing Stranger 1 compared to the empty cup.

The protocol for defensive burying was adapted from a well-defined protocol [28]. Mice were placed in a rat-sized tub cage filled with 5 cm of corn cob bedding for 5 min with 20 marbles in a 5 × 4 array, during which time the time spent digging was recorded. Digging was defined as coordinated movements of fore or hind limbs that displaced the bedding.

A combined object displacement and novel object recognition task was performed to assess spatial and object recognition memory. This task consisted of three trials, each lasting 5 min, with an inter-trial interval of 15 min. In the first trial, two of the same objects were placed in the open field arena, one in the middle of the front left quadrant, and one in the middle of the front right quadrant. In the second trial, one of these objects had its position moved to the back quadrant of the same side (left or right) of the arena, while the other remained in the same location as the first trial. The ability to discriminate the novel location of the object was assessed as the percentage of time spent with object in novel location (displaced object) compared to object in the same location as the previous trial. In the third trial, the object that was previously moved to a novel location was replaced by a novel object, while the other object remained the same and in the same location. Novel object recognition was assessed by the percentage of time spent with novel object compared to the object used for the previous two trials (familiar object).

In the Y-maze for spontaneous alternation task, mice were placed in a Y-shaped maze consisting of three arms, as done previously [19]. Animals were allowed to freely explore the arms for 3 min, and the number of arm entries was recorded. The order of arm entries was also manually assessed, and percent alternation was calculated [# alternations/(# arm entries − 2) × 100]. One alternation consists of a mouse going down each of the three arms before returning to a previously visited arm. In normal exploration, mice should alternate traversing down arms in a circular pattern rather than repeatedly going down the same arms, based on the natural tendency of mice to explore novel environments. Chance performance for continued alternation is 22.2%.

The Barnes maze test was performed as previously described [19]. Briefly, testing was performed on 5 consecutive days, with two trials per day separated by a 15 min inter-trial interval. Mice were placed onto the center of the maze at the beginning of each trial, then allowed to explore until the hole with the escape box was found and entered, or a maximum of 5 min.

### Physiological and neuropathological measures

Following the 8-month exercise intervention period and completion of behavioral testing, mice were euthanized under deep anesthesia with 2.5% avertin. Cardiac puncture was performed to collect blood, which was allowed to clot at room temperature for 30 min, spun at 2000×*g* for 10 min, and serum was collected and stored at − 80 °C until used in assays. Following blood collection, mice were perfused with saline. Brains were bisected along the midline and prepared for subsequent pathological analyses. One hemisphere was placed in 70% ethanol and subsequently paraffin embedded for immunohistochemistry, while the other was flash frozen in liquid nitrogen for quantitative polymerase chain reaction. Serum samples were also analyzed using a commercially available ELISA for corticosterone according to the manufacturer’s instructions (Cayman Chemical). Absorbance was recorded using a plate reader (Spectramax).

#### Enzyme-linked immunosorbent assay (ELISA) for Aβ species

ELISAs were performed to quantify whole forebrain levels of soluble (TBS) and insoluble (guanidine) forms of Aβ40 and Aβ42 in Tg-SwDI mice (Sed *n* = 9, 1 h n = 9, 3 h *n* = 8, 12 h *n* = 8). The brains of WT mice do not display amyloid pathology, as assessed by ELISA, and therefore were not analyzed. Methods for this have been previously described [10].

#### Histology

Brain hemispheres embedded in paraffin were sectioned at 10-μM thickness and mounted on glass slides. Paraffin was removed from sections by immersion in xylene (3 × 5 min) and rehydrated in decreasing concentrations of ethanol (100%, 95%, 70%, 50%, 0% at 5 min each). Staining and analysis for total and vascular fibrillar Aβ burden were then performed as below.

### Total Aβ staining and analysis

Histological analysis was performed to qualitatively assess total Aβ burden in Tg-SwDI mice to confirm results of the ELISAs. Deparaffinned and rehydrated sections were washed in PBS for 5 min. Tissue was permeabilized and non-specific antibody binding was blocked by treatment with 0.3% Triton X-100 in 1:10 SuperBlock blocking buffer (Thermo) in PBS for 30 min at room temperature. The tissue was then incubated with primary antibody (1:200 rabbit polyclonal anti-Aβ 1-28; in 0.1% Triton X-100 in 1:10 SuperBlock blocking buffer in PBS overnight). The next day, slides were washed 3 × 5 min with distilled water, then incubated for 2 h at room temperature with secondary antibody (1:1000 Alexa Flour 488-conjugated anti-rabbit IgG) in 0.1% Triton X-100 in 1:10 SuperBlock blocking buffer in PBS. Slides were rinsed 3× with distilled water then 2× with 70% EtOH, then washed for 5 min in distilled water. The tissue was mounted in Glycerol/PBS (25:10) mounting media to preserve fluorescence. Sections were imaged using an Olympus BX60 microscope with an attached Olympus Dp72 camera. Images were collected from each section at × 10 magnification.

### Thioflavin-S and collagen IV staining and analysis

Histological analysis was performed to assess regional fibrillar amyloid deposition around the cerebral vasculature (CAA pathology) using procedures previously described [10–14] Since WT mice do not exhibit measurable CAA pathology, only Tg-SwDI mice were used in this analysis (Sed *n* = 9, 1 h *n* = 9, 3 h *n* = 8, 12 h *n* = 6). Deparaffinned and rehydrated sections were washed in PBS for 5 min, followed by a 5 min incubation with proteinase K (0.02 mg/mL, IBI Scientific) in PBS for antigen retrieval, then rinsed in distilled water 5 × 1 min. Tissue was permeabilized and non-specific antibody binding was blocked by treatment with 0.3% Triton X-100 in 1:10 SuperBlock blocking buffer (Thermo) in PBS for 30 min at room temperature. The tissue was then incubated with primary antibody (1:100 rabbit polyclonal anti-collagen type IV; Cat # PA1-36063; Thermo Fisher) in 0.1% Triton X-100 in 1:10 SuperBlock blocking buffer in PBS overnight. The next day, slides were washed 3 × 5 min with distilled water, then incubated for 2 h at room temperature with secondary antibody (1:1000 Alexa Flour 594-conjugated anti-rabbit IgG) in 0.1% Triton X-100 in 1:10 SuperBlock blocking buffer in PBS. Slides were rinsed with distilled water, then stained for Thioflavin-S (0.0125% Thioflavin-S in 50% EtOH/PBS) by incubating for 15 min at room temperature. Slides were rinsed 3× with distilled water then 2× with 70% EtOH, then washed for 5 min in distilled water. The tissue was mounted in Glycerol/PBS (25:10) mounting media to preserve fluorescence. Sections were imaged using an Olympus BX60 microscope with an attached Olympus Dp72 camera. Images from the cortex, subiculum, and thalamus were collected from each section at × 40 magnification. Using NIH ImageJ software, an appropriate threshold was set for each stain and the percent area occupied with positive stain was quantified. Fibrillar amyloid accumulation around the vasculature was assessed in the cortex, subiculum, and thalamus. These regions were chosen as they have been previously shown to accumulate fibrillar amyloid and are involved in several of the behavioral tasks used in the current study. Vascular amyloid deposition (percentage of blood vessel coverage with fibrillar amyloid) was calculated by [(ThioflavinS+ stain/Collagen IV+ stain) × 100].

#### Quantitative polymerase chain reaction (qPCR) for inflammatory cytokines

Brain hemispheres that were flash frozen in liquid nitrogen were homogenized, and RNA was isolated from this forebrain homogenate using a commercially available kit (PARIS Kit #AM1921, Ambion by Life Technologies) and treated with DNase. These tissues were collected following the 8-month exercise intervention period and completion of behavioral testing. Samples from each treatment group were analyzed (WT Sed *n* = 6, Tg-SwDI Sed *n* = 8, Tg-SwDI 1 h *n* = 9, Tg-SwDI 3 h *n* = 8, Tg-SwDI 12 h *n* = 7). RNA concentration was checked to ensure sample quality. All samples were of adequate RNA concentration for use in subsequent analysis and were reverse transcribed into cDNA using dT primers using a commercially available kit, then aliquoted. qPCR was performed to attain expression levels of inflammatory cytokines tumor necrosis factor alpha (TNF-α), interleukin-6 (IL-6), interleukin-1 (IL-1), interleukin-1 beta (IL-1β), and interferon gamma (IFN-ɣ), with beta-actin used as the housekeeping gene control. Statistical analyses using the delta-delta C_T_ method were performed on ΔCt values, and data was plotted as fold change from WT sedentary mice.

### Statistical analyses

One-way ANOVA’s were performed to determine differences between groups for serum corticosterone concentration, behavior, and pathology measures. Two-way repeated measures ANOVAs were performed to assess differences in groups over time for running parameters, food intake, and body weight. Two-way repeated measures ANOVAs were also performed to assess differences in exploration of objects (during the object displacement and novel object recognition task) or cups (during social interaction) between treatment groups, as well as group differences in Barnes maze performance over time. Analyses were performed using Statistica and SigmaPlot/Stat, and significance was set at alpha = 0.05.

## Results

### Running parameters

The number of rotations performed by each mouse was recorded with computer software in 1-min bins during daily exercise sessions. The total number of rotations performed was summed during each session as a measure of running volume. Additionally, two measures of running quality, or “intensity”, were calculated. Breaks per hour was calculated to determine the number of 1-min bins per hour during which the mouse was not running. A “break” was defined as a 1-min bin in which the animal performed fewer than five wheel rotations. Running speed (rotations/minute) was calculated by averaging the number of rotations performed in 1-min bins that were not counted as a “break”, essentially averaging the number of rotations performed per minute when the number of rotations performed in that bin was greater than five.

#### Rotations

**Fig. 1:**
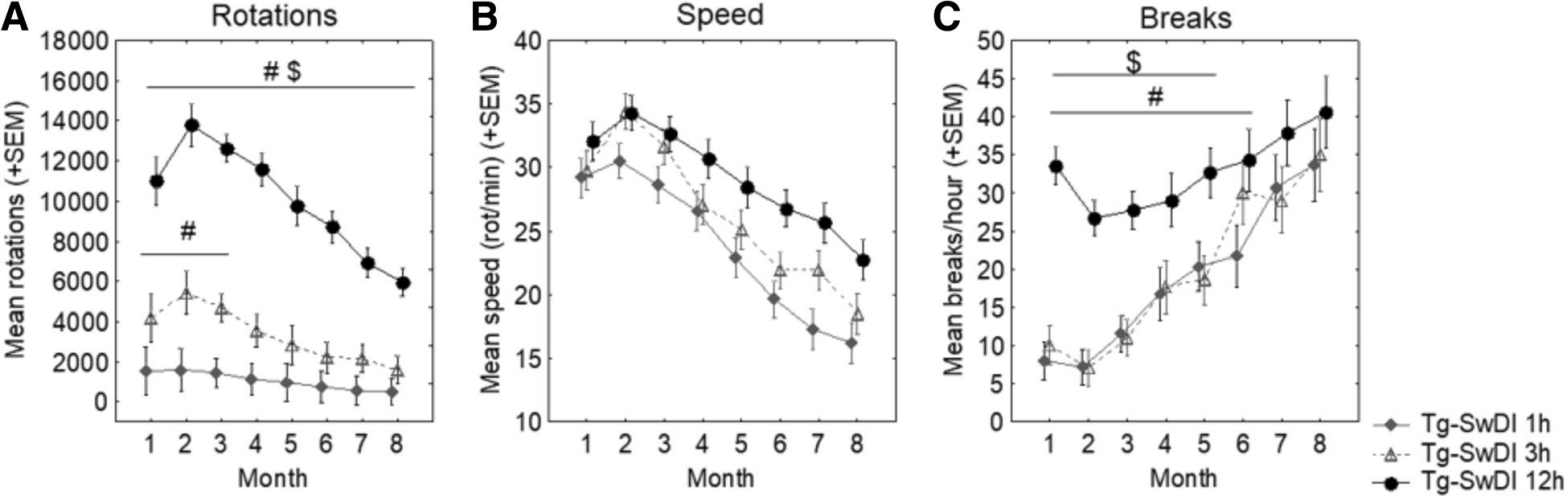
Monthly wheel running parameters over the course of the 8-month exercise intervention period. **a** The number of rotations performed by each mouse was recorded with computer software in 1-minute bins during each exercise session. The total number of rotations performed was summed during each session as a measure of running volume. **b** Running speed (rotations/minute) was calculated by averaging the number of rotations performed in 1-min bins that were not counted as a “break,” essentially averaging the number of rotations performed per minute when the number of rotations performed in that bin was greater than five. **c** Breaks per hour was calculated to determine the number of 1-min bins per hour during which the mouse was not running. A “break” was a 1-min bin in which the animal performed less than five wheel rotations. *#p < 0.05* versus *Tg-SwDI 1 h; $p < 0.05* versus *Tg-SwDI 3 h*

Average rotations performed per exercise session, over the course of the intervention period, was analyzed by month (Fig. 1a). Overall, there was an increase in rotations performed with increasing access to a running wheel (12 h > 3 h > 1 h; *p* < 0.05 for all). Additionally, there was an overall increase in the number of rotations performed from the first to the second month of the intervention period, then running dropped off steadily in subsequent months. Tg-SwDI 3 h mice ran more than Tg-SwDI 1 h mice during all months; however, this was only significant in months 1–3 (*p* < 0.05 for all). Tg-SwDI 12 h mice ran more than both 1 h and 3 h mice during all months of the intervention period (*p* < 0.05 for all).

#### Speed (rotations per minute)

Running speed over the course of the intervention period was analyzed by month (Fig. 1b). Overall, running speed increased with increasing access to a running wheel (3 h > 1 h (*p* = 0.10); 12 h > 1 h (*p* = 0.001); 12 h > 3 h (*p* = 0.060)). Additionally, there was an overall increase in running speed from the first to the second month of the intervention period, then running speed dropped off steadily in subsequent months.

#### Breaks per hour

Average breaks taken per hour during exercise sessions over the course of the intervention period were analyzed by month (Fig. 1c). Overall, the number of breaks taken per hour was higher in Tg-SwDI 12 h mice compared to Tg-SwDI 1 h and 3 h mice (*p* < 0.05 for both). Additionally, there was an overall decrease in breaks per hour taken from the first to the second month of the intervention period, then breaks increased steadily in subsequent months. Tg-SwDI 12 h mice took more breaks than both 1 h (months 1–6) and 3 h (month 1–5) mice (*p* < 0.05 for all).

### Physiological measures

#### Body weight and food intake

**Fig. 2:**
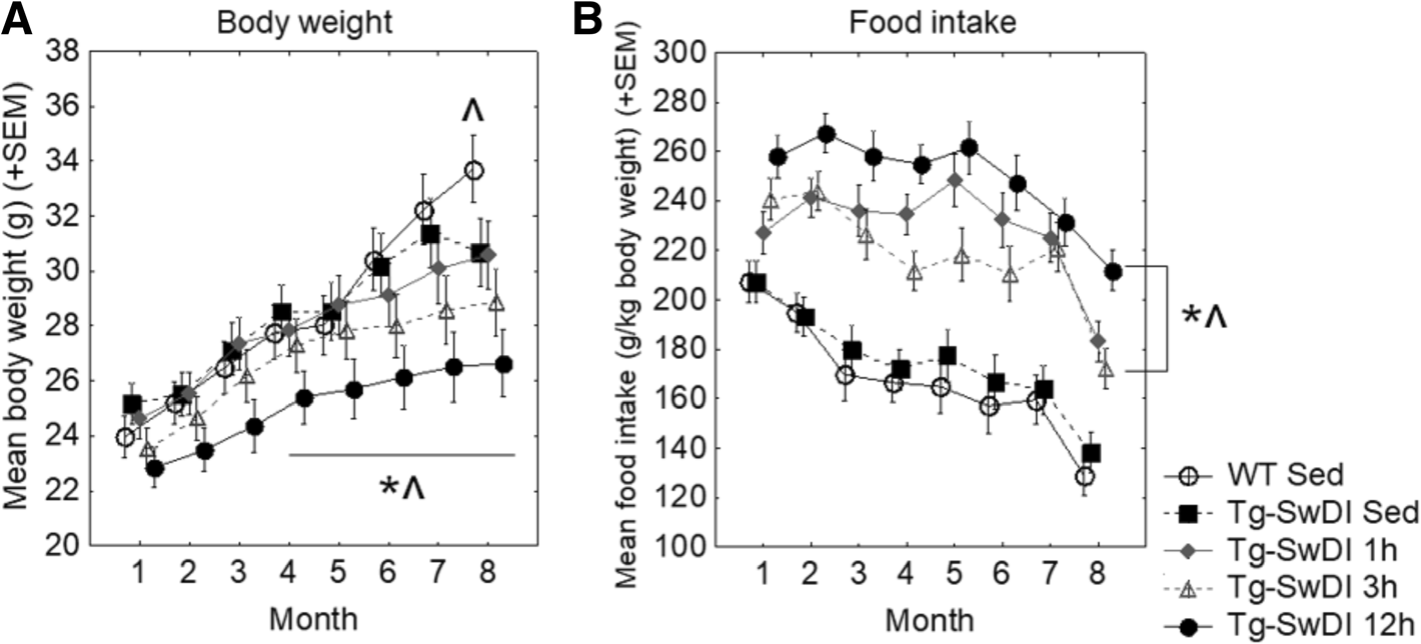
Body weight and food intake over the course of the exercise intervention period. **a** Monthly body weights. **b** Average daily food intake by month of exercise intervention. *p < 0.05 versus *WT sedentary; ^p < 0.05* versus *Tg-SwDI sedentary*

Body weight (Fig. 2a) and food intake (Fig. 2b) were assessed throughout the course of the intervention period and analyzed by month. All mice gained weight over the course of the intervention period. Tg-SwDI sedentary mice weighed similarly to WT sedentary controls, except in the last month of the intervention, when Tg-SwDI sedentary weight gain dropped off compared to WT (*p* < 0.05). Within Tg-SwDI mice, only 12 h mice showed significant reductions in body weight compared to sedentary mice, and this occurred in months 4–8 (*p* < 0.05 for all).

While there was no difference between Tg-SwDI sedentary and WT sedentary controls, all exercised Tg-SwDI mice ate more than both sedentary groups (*p* < 0.05 for all). Additionally, Tg-SwDI 12 h mice ate more than Tg-SwDI 1 h (*p* = 0.074) and 3 h (*p* < 0.05) mice overall.

#### Serum corticosterone

There was a non-significant trend of the Tg-SwDI 1 h mice having higher corticosterone levels compared to all other groups (data not shown). No other exercise groups showed notable deviation from controls.

### Behavioral performance

**Fig. 3:**
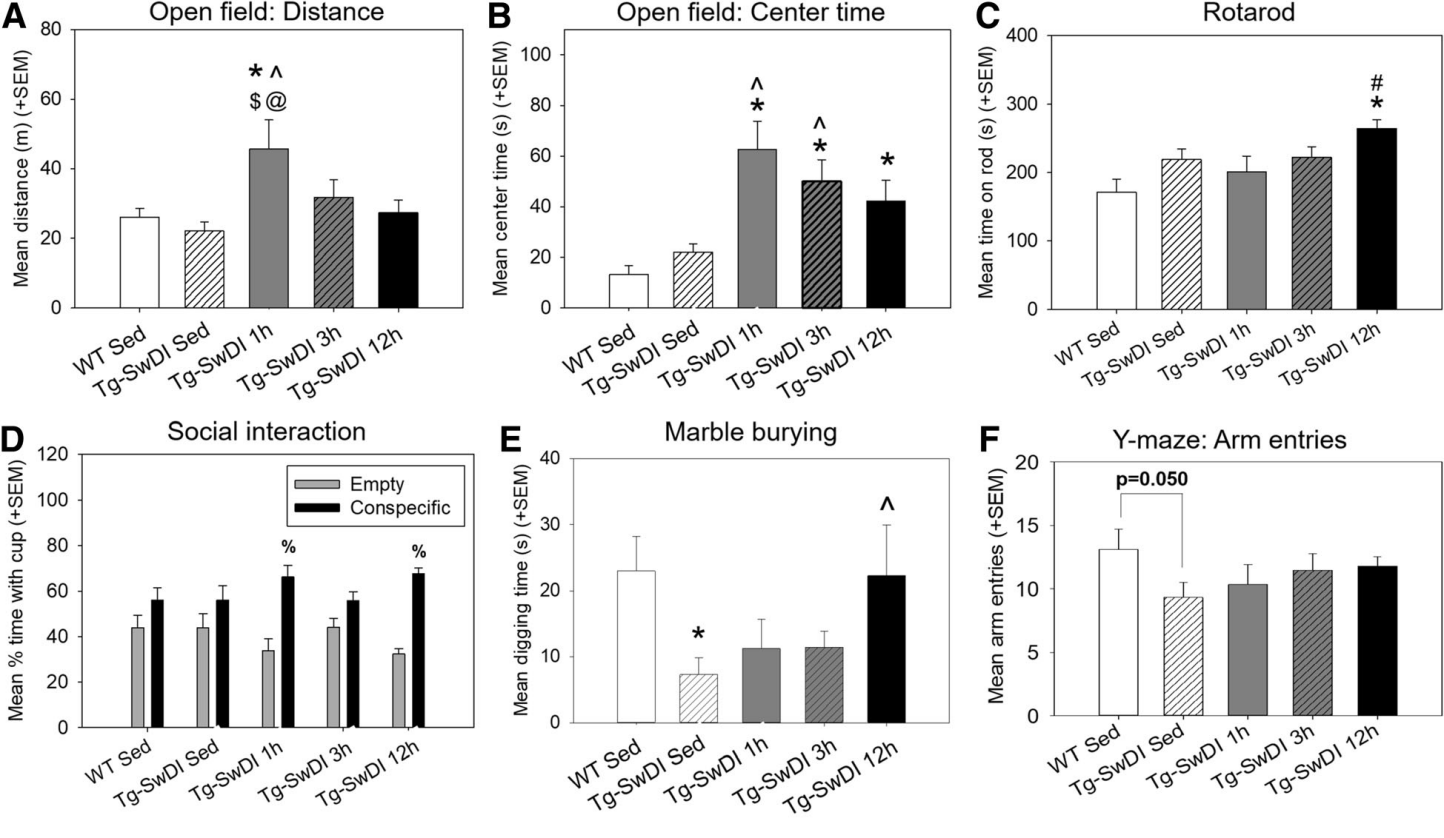
Performance on behavioral tasks assessing motor function, exploratory behavior, and temperament. **a** Distance traveled in the open field arena during the open field test, in meters. **b** Time spent in the center of the arena during the open field test, in seconds. **c** Rotarod performance, as measured by time spent on the rod, in seconds (max time = 300 s). **d** Percentage of time spent with empty versus conspecific cup in the social interaction test. **e** Time spent digging in the defensive burying task, in seconds. **f** Exploration in the Y-maze task, as measured by number of arm entries. *p < 0.05 versus *WT sedentary; ^p < 0.05* versus *Tg-SwDI sedentary, #p < 0.05* versus *Tg-SwDI 1 h; $p < 0.05* versus *Tg-SwDI 3 h; @p < 0.05* versus *Tg-SwDI 12 h; %p < 0.05 more time spent with conspecific cup* vs. *empty cup*

**Fig. 4:**
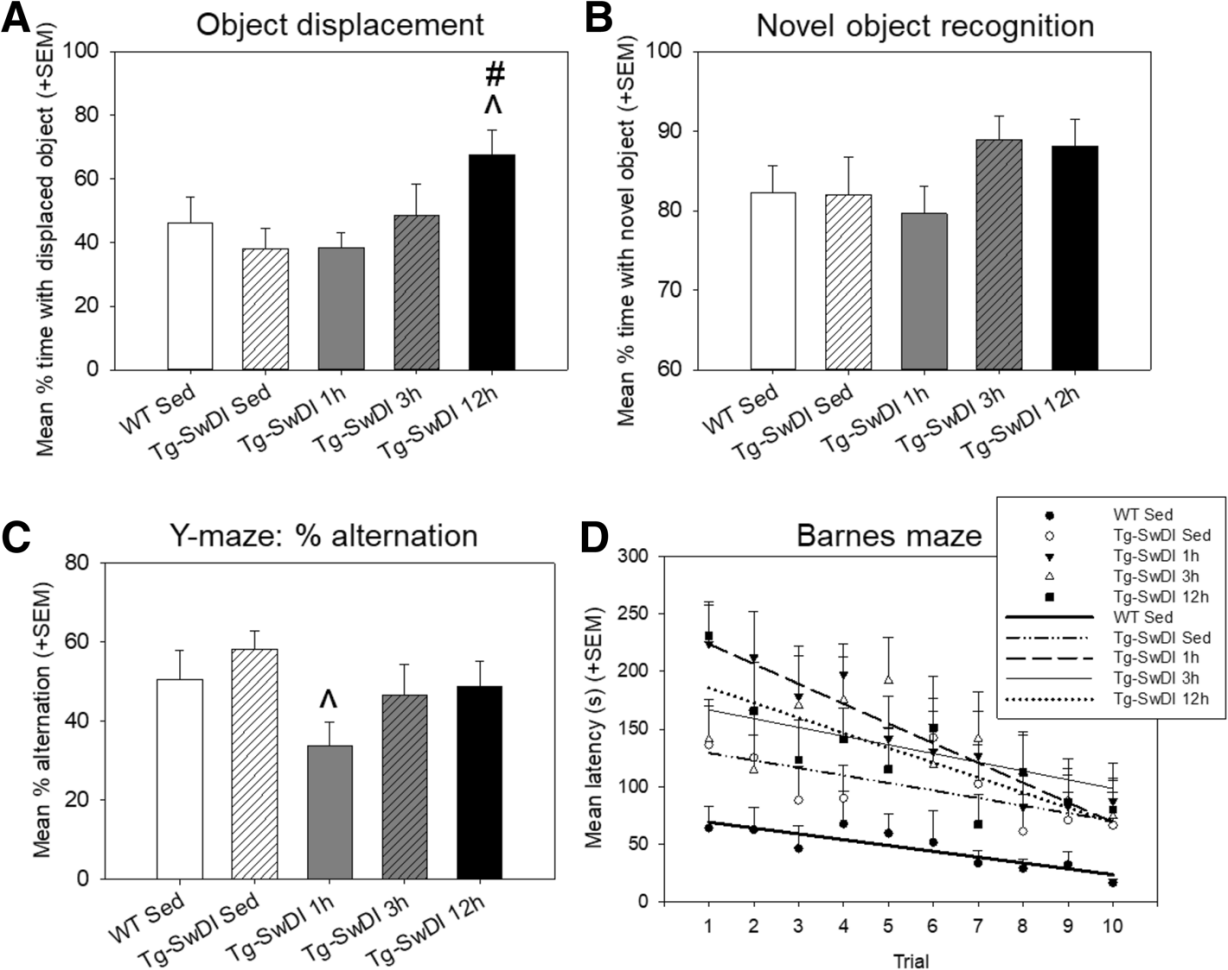
Performance on cognitive tasks of learning and memory. **a** Performance on the object displacement task, as measured by percentage of time spent with the displaced object. **b** Performance on the novel object recognition task, as measured by percentage of time spent with the novel object. **c** Performance on the Y-maze task, as measured by percent alternation between arms. **d** Performance on the Barnes maze task by trial, as measured by latency to find the escape hole (max time = 300 s). Data points for each group were fit with a linear line to quantify the rate of learning. *^p < 0.05* versus *Tg-SwDI sedentary, #p < 0.05* versus *Tg-SwDI 1 h*

We hypothesized that exercise’s beneficial behavioral alterations might be observed in either a restorative “rescue” pattern, in which an apparent impairment in a measure of performance between Tg-SwDI versus WT animals is rescued by exercise, or where exercise altered a behavioral measure not sensitive to genotype differences in the mice (“exercise-induced enhancement”). This latter effect is, at best, interpreted as showing that the Tg-SwDI mice were not limited due to their pathology from demonstrating an exercise-induced alteration in behavior. Figure 3 shows measures from tasks reflecting motor, exploratory, and temperamental behaviors, and Fig. 4 shows measures from tasks assessing different aspects of learning and memory.

#### Motor, exploratory, and temperament measures

Several of the behavior measures in Fig. 3 showed clear exercise-induced enhancement patterns, with volume (i.e., change greatest in 12 h group) or intensity-dependent effects (i.e., change greatest in the 1 h or 3 h groups). There was no difference between Tg-SwDI sedentary and WT sedentary controls in either distance traveled in the open field (a measure of overall activity) or time in the center of the arena (an inverse measure of anxiety-like behavior). Exercise increased both measures in an intensity-dependent manner for distance and center time. Tg-SwDI 1 h mice traveled a greater distance, and all Tg-SwDI exercise groups spent more time in the center of the arena, compared to both sedentary groups (*p* < 0.05 for all except Tg-SwDI 12 h vs. Tg-SwDI sedentary (*p* = 0.073)).

In the rotarod, there was again no significant difference in performance between WT sedentary controls and Tg-SwDI sedentary mice, though Tg-SwDI 12 h mice performed better than Tg-SwDI and WT sedentary controls (*p* < 0.05 for both). This pattern of exercise-induced enhancement was again evident in the social interaction test, where there was no difference in preference for conspecific cup vs. empty cup for the two sedentary groups, preference for exploration of the conspecific cup was detected for two of the exercise groups (1 h and 12 h, *p* < 0.05).

The two measures that appear to show a “rescue” type pattern were digging time in the marble-burying task and the number of arm entries in the Y-maze task. In these measures, the WT sedentary controls had greater digging time and greater Y-maze arm entries compared to Tg-SwDI sedentary mice (*p* < 0.05). In the marble-burying task, the Tg-SwDI 12 h exercise group exhibited greater digging time than Tg-SwDI sedentary mice (*p* < 0.05) and had similar performance to WT sedentary mice. In the Y-maze task, the number of arm entries performed by the Tg-SwDI exercise groups was not significantly different than the sedentary WT group (*p* > 0.05 for all).

#### Learning and memory measures

Object displacement, novel object recognition (NOR), and Y-maze % alternation measures were not sensitive to differences in sedentary WT vs. sedentary Tg-SwDI groups, though some modest benefits of exercise in Tg-SwDI mice were apparent on some of these measures (Fig. 4). The Tg-SwDI 12 h mice exhibited increased performance compared to both Tg-SwDI sedentary and 1 h mice on the object displacement task (*p* < 0.05 for both). Additionally, there was a slight trend toward improved performance on the NOR task; lack of significance could have resulted from a ceiling effect of performance, since even the sedentary groups spent more than 80% of time exploring the novel compared to the familiar object.

In our hands, the Barnes maze has been the most reliable behavioral assay at showing spatial learning and memory deficits (increased latency to find the escape box) of Tg-SwDI as compared to WT controls [23, 24, 29, 30]. This effect is again evident here, with all Tg-SwDI groups having an increased latency to find compared to the WT sedentary group (*p* < 0.05 for all except Tg-SwDI sedentary *p* = 0.109). Slopes of linear best fit, representative of rate of learning, did not significantly differ between groups (*p* > 0.05).

### Pathology

#### Cerebral Aβ burden

**Fig. 5:**
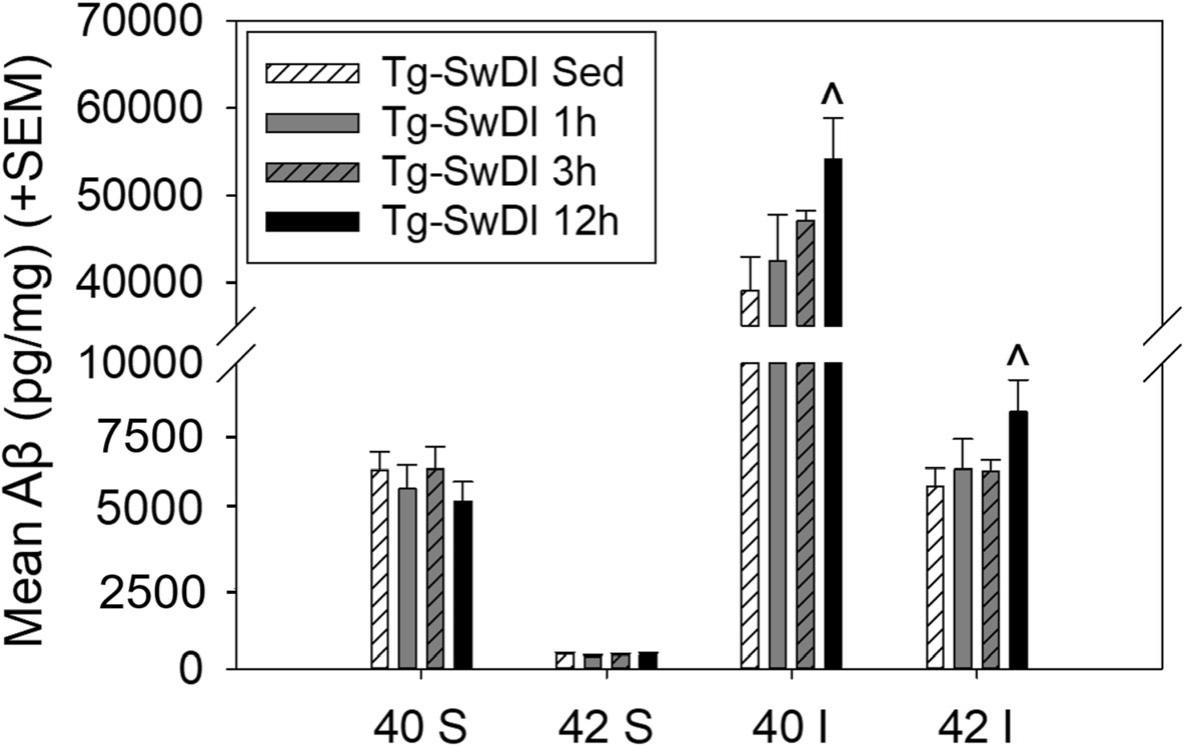
Levels of beta-amyloid species in forebrain homogenate of Tg-SwDI mice by ELISA. Brains were collected following the 8-month exercise intervention period and completion of behavior testing. Forebrain homogenate was assayed for levels of beta-amyloid (Aβ) 40 and 42 in soluble (S) and insoluble (I) fractions. *^p < 0.05* versus *Tg-SwDI sedentary*

Effects of exercise on four species of Aβ were tested using ELISAs (40S, 42S, 40I, and 42I) (Fig. 5). WT mice were not included in the analysis, since only Tg-SwDI mice exhibit measurable Aβ accumulation. Pairwise comparisons revealed that 12 h exercise mice exhibited a greater amount of both insoluble Aβ species (40I and 42I) compared to sedentary mice (*p* < 0.05 for both).

#### Vascular amyloid burden

**Fig. 6:**
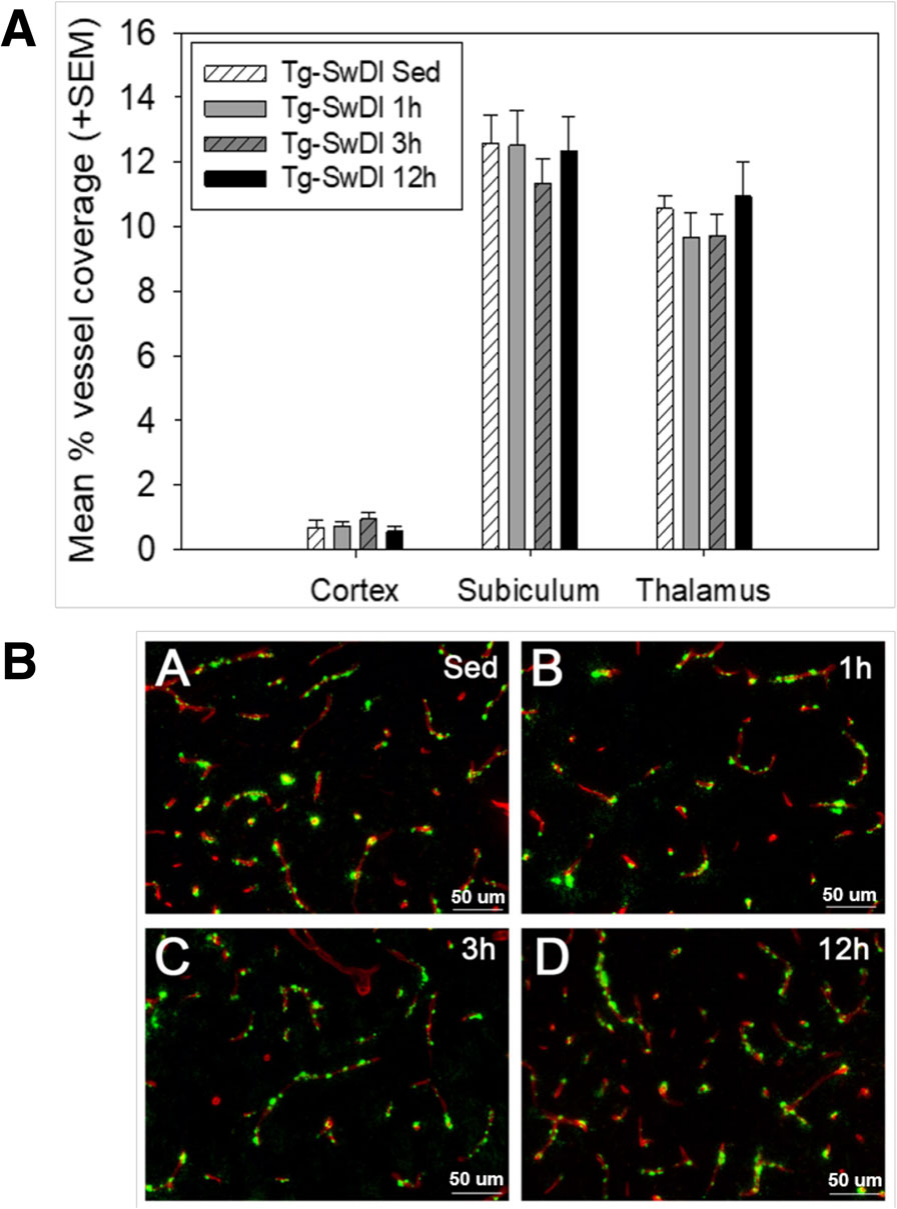
Quantitative and histological illustration of vascular amyloid burden. **a** Vascular amyloid burden in Tg-SwDI mice, represented as percent of the vasculature covered by fibrillar amyloid in the cortex, subiculum, and thalamus. This measure was calculated as [(ThioflavinS^+^ stain/Collagen IV^+^ stain) × 100]. WT mice were not included in this analysis, as only Tg-SwDI mice exhibit measurable amyloid pathology. Brains were collected following the 8-month exercise intervention period and completion of behavior testing. *^p < 0.05* versus *Tg-SwDI sedentary*. **b** Representative images of histological staining from thalamus (scale bars = 50 μm) for fibrillar Aβ (Thioflavin-S; green) and blood vessels (collagen IV; red) in sagittal sections from Tg-SwDI mouse brains

Effects of exercise on vascular fibrillar amyloid deposition in several brain regions of interest (cortex, subiculum, and thalamus) (Fig. 6). WT mice were not included in these analyses, as only Tg-SwDI mice exhibit measurable amyloid pathology. Exercise had no significant effect on CAA pathology in any region analyzed. Representative images of the thalamic region are shown in Fig. 6b.

#### Inflammatory cytokine expression

**Fig. 7:**
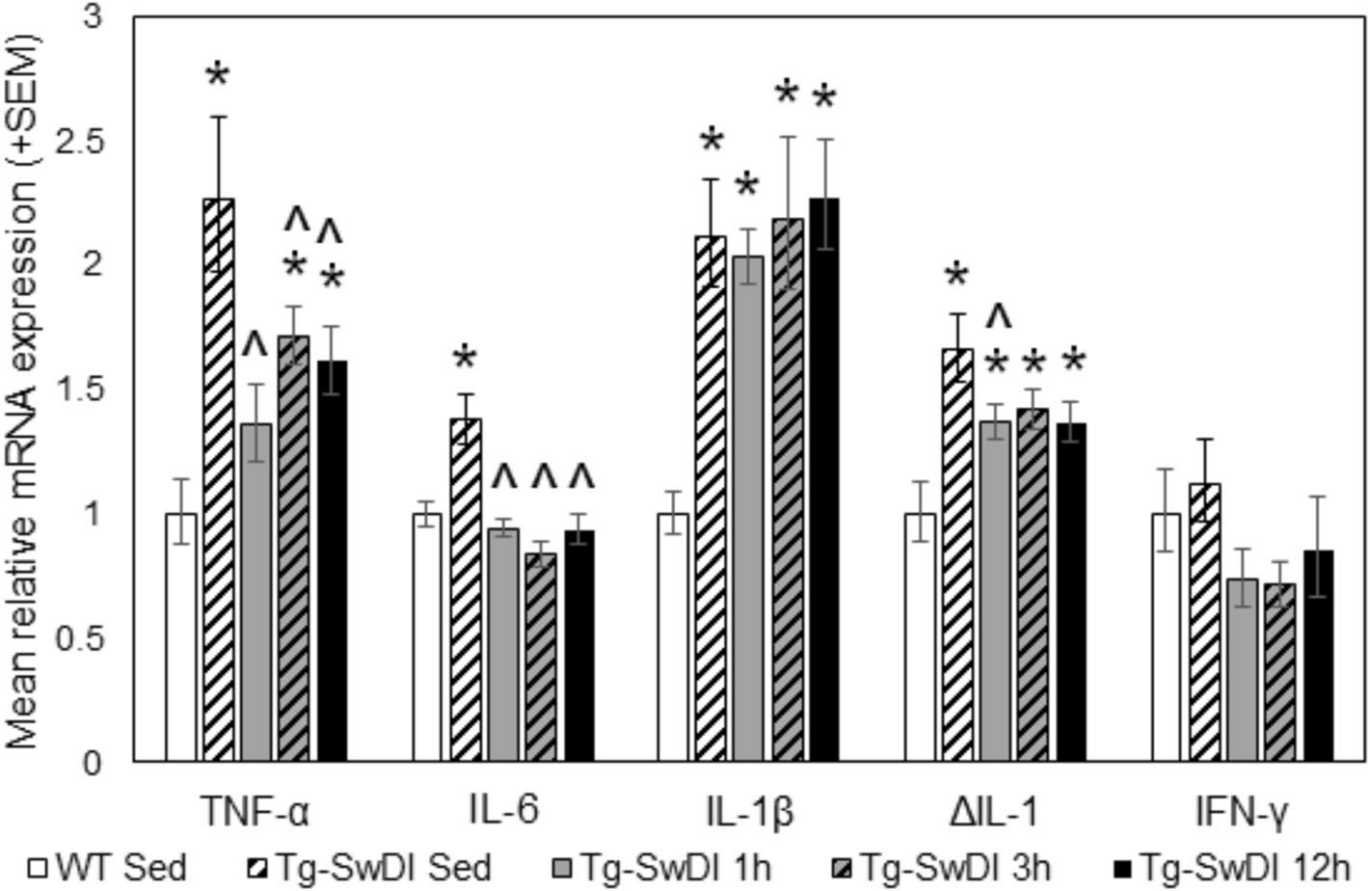
Quantitative polymerase chain reaction (qPCR) results showing inflammatory cytokine expression. Expression levels of TNF-α, IL-6, IL-1β, IL-1, and IFN-γ were assessed in forebrain homogenate, collected following the 8-month exercise regimen and behavior testing. Statistical analyses were performed on ΔCt values (one-way ANOVA (between-subjects factor: group)), and data are plotted as fold change from WT sedentary mice. *p < 0.05 vs. *WT sedentary controls. ^p < 0.05* vs. *Tg-SwDI sedentary mice*

Expression of inflammatory cytokines TNF-α, IL-6, IL-1β, IL-1, and IFN-γ were assessed in forebrain homogenate using qPCR (Fig. 7). Statistical analyses were performed on ΔCt values using the delta-delta C_T_ method, and data plotted as fold change from WT sedentary mice. Compared to WT sedentary controls, Tg-SwDI sedentary mice had increased expression of TNF-α, IL-6, IL-1β, and IL-1 (*p* < 0.001 for all), while there was no difference in IFN-γ expression. All exercise doses reduced TNF-α expression compared to Tg-SwDI sedentary mice (*p* < 0.05 for all), but 3 h and 12 h Tg-SwDI mice still had greater expression levels than WT sedentary controls (*p* < 0.05 for both). All exercise doses also reduced IL-6 expression compared to Tg-SwDI sedentary mice (*p* < 0.05 for all), becoming indistinguishable from levels of WT sedentary controls (*p* > 0.05 for all). All exercise doses reduced IL-1 expression compared to Tg-SwDI sedentary mice (1 h *p* = 0.031, 3 h *p* = 0.092, 12 h = 0.055); however, these comparisons did not all reach statistical significance. Additionally, IL-1 expression in all Tg-SwDI exercise groups was still significantly elevated above WT sedentary control levels (*p* < 0.05 for all). In Tg-SwDI mice, exercise had no significant effect on expression levels of IL-1β or IFN-γ.

## Discussion

This study is the first examination of the effects of cardiovascular exercise (CVE) on cerebral amyloid angiopathy (CAA) pathology and behavioral deficits in a rodent model. As a vascular pathology, it was hypothesized that CAA might be especially susceptible to beneficial effects of CVE, or, conversely, CVE could be detrimental due to added stress on an already compromised cerebrovascular system in CAA. We utilized the Tg-SwDI mouse, a well-characterized model of CAA, and examined the physiological, neuropathological, and behavioral effects of 8 months of voluntary CVE, beginning at age of 3.5–4 months of age, modeling a lifetime of CVE in humans. We also varied the length of daily access to a running wheel to determine whether the effects of exercise were “dose-dependent.”

### Varying access time to running wheels produced differences in running volume and intensity, and dose-dependently altered physiology

There has been debate in the literature regarding the relative efficacy of high-volume versus high-intensity CVE regimens to produce health benefits. Some studies suggest that longer-duration exercise is more beneficial compared to regimens shorter in duration [31], while others suggest that only small amounts of exercise are necessary to see significant health benefits [32–34]. There is evidence that shorter duration, high-intensity regimens are equally beneficial to longer-duration/lower-intensity regimens with regard to cardiovascular and metabolic function and musculoskeletal benefits [35–38], while others have found greater benefits of lower intensity exercise, such that long-term lower intensity exercise had increased efficacy compared to high-intensity exercise for improving cognition, enhancing adult hippocampal neurogenesis, and altering the transcriptome of the hippocampus, including genes involved in lipid metabolism, protein synthesis, and the inflammatory response [39, 40]. Therefore, creating exercise regimens with varying duration/volume and intensity in mice allows assessment of the effects of voluntary exercise regimens adopted by people that incorporate varying levels of exercise intensity and volume. Although studies have previously utilized varying duration and intensity of exercise using forced exercise (e.g., treadmill running), our study creates different patterns of exercise using voluntary wheel running, which has the benefit of avoiding the confounding element of stress and not simply programming the exercise as a linear “dose” of continuous exercise volume [26].

Most studies that utilize wheel-running exercise provide unlimited wheel access. As nocturnal animals, mice will not usually run much during their light (sleep) cycle. Therefore, our Tg-SwDI 12 h group is comparable to unlimited wheel access groups in other studies. The shorter access periods produced qualitatively distinct running patterns compared to Tg-SwDI 12 h mice, needed to model exercise volume and intensity variation. Both the 1 h and 3 h groups demonstrated high-intensity running patterns in that they took few breaks from running within each session. Both also showed reduced running speed (rotations/min) as compared to 12 h group mice, perhaps as a means to sustain longer bouts of running. Running volume was proportionately greater in the 3 h group as compared to the 1 h group, making it an intermediate condition that combined substantial exercise intensity and volume. Our results are comparable to those seen by providing the same running wheel access conditions to healthy C57BL/6 mice of similar age, which found similarly increased running volume and altered measures of running intensity with increased access to a running wheel [19]. Our findings therefore do not reflect running patterning unique to this mouse model.

We found that exercise had no significant effect on serum corticosterone levels, suggesting that the exercising mice were metabolically well adapted to the running paradigm and not experiencing enhanced levels of physiological stress. Exercise did, however, dose-dependently increase food intake in Tg-SwDI mice, likely to compensate for energy deficits resulting from exercise, in a manner similarly shown in healthy C57BL/6 mice of similar age [19]. Increased length of access to a running wheel resulted in reduced body weight, but this was only significant for the 12 h group and was only seen following 4 months of exercise intervention. Previously, we found that in healthy C57BL/6 mice of similar age, all of these exercise doses attenuated body weight [19].

### Exercise alters motor function, sociability, and temperament in Tg-SwDI mice but has only modest effect on learning and memory

Measures in several tasks reflective of general activity, motor competence, anxiety-like behavior, sociability, and memory for a displaced object, showed exercise-induced enhancement, such that exercise improved performance over sedentary levels when a deficit in Tg-SwDI sedentary versus WT sedentary mice did not exist. In Tg-SwDI mice, trends of exercise-induced increases in locomotor behavior in the open field and increased social interaction with exercise (particularly 1 h and 12 h groups) were quite similar to those seen utilizing the same exercise regimens in C57BL/6 mice [19]. There were also trends of 3-h and 12-h exercise mice exhibiting enhanced object recognition memory; this was not significant, possibly due to a ceiling effect, similar to results seen in our previous study using the same exercise regimens in C57BL/6 mice [19]. Taken together, these findings suggest that the Tg-SwDI mice were not limited, due to their pathology, from demonstrating exercise-induced alterations in behavior that are similar to healthy WT mice. One task that did show differences in susceptibility to exercise between Tg-SwDI mice and previously tested C57BL/6 mice is the rotarod. While all exercise regimens augmented performance in C57BL/6 mice [19], only 12-h exercise significantly increased performance in Tg-SwDI mice, suggesting that perhaps motor competence is less sensitive to exercise training in this strain compared to healthy mice.

In some tasks, behavior of Tg-SwDI sedentary mice differed from WT sedentary mice. Effects of exercise were considered to be restorative (“rescue” pattern) if exercise altered Tg-SwDI behavior to be more similar to WT sedentary controls. Tg-SwDI sedentary mice exhibited reduced digging behavior and decreased exploration of Y-maze arms compared to WT sedentary controls. These differences are consistent with the previously observed tendency of Tg-SwDI mice to exhibit slower rates of exploration of stimulus objects or maze arms when multiple options are available, thought to represent a form of perceptual slowing characteristic of CAA [18]. One significant instance of a restorative “rescue” pattern was seen in the Y-maze task, such that marginally reduced exploratory behavior in the Y-maze exhibited by Tg-SwDI sedentary versus WT sedentary mice was not seen in Tg-SwDI exercise groups. Additionally, a “rescue” pattern of exercise increasing marble burying in Tg-SwDI mice was observed, while previously we also found that exercise increased marble burying in C57BL/6 mice albeit not to a significant degree [19].

While sedentary Tg-SwDI mice exhibited impaired performance in the Barnes maze (longer latency to find the escape hole) compared to sedentary WT mice, no restorative effects in learning and memory were observed in the exercise groups. In fact, Tg-SwDI exercise mice tended to take longer to find the escape hole compared to Tg-SwDI sedentary mice in the first few trials though showed comparable learning rates to the Tg-SwDI sedentary mice overall. While exercise has generally been shown to improve spatial learning and memory in healthy mice and mouse models of AD [41–43], our previous study using the same exercise regimens in healthy C57BL/6 mice of similar age found similar trends, with some exercise groups showing an increased latency to find the escape hole in the Barnes maze task [19]. This may be attributable to exercise mice exhibiting a reduced anxiety response in bright open spaces (as is the Barnes maze), attenuating the motivation to find the escape hole. This possibility is supported by Tg-SwDI exercise mice displaying increased time in the center of the open field arena, as was also shown in all exercised C57BL/6 mice in our previous study [19].

### CVE reduces neuroinflammation but does not reduce amyloid pathology

Exercise did not significantly affect vascular amyloid deposition (CAA) in any of the brain regions studied. Surprisingly, high-volume exercise (12 h group) increased levels of insoluble Aβ without having a significant effect on soluble Aβ. As insoluble Aβ made up the significant majority of total amyloid burden, these findings were supported by qualitative analysis of total Aβ histology. Others have found that exercise reduces Aβ pathology [9, 44]; however, Jankowsky et al. found that 6 months of housing in an enriched enrichment, which included an exercise component, increased Aβ levels in APPswe and APPswe/PS1dE9 mice [45, 46]. These studies did not differentiate between soluble and insoluble species, though given the abundance of insoluble over soluble Aβ, overall differences likely suggest changes to insoluble levels. Similar to the current study, these increases in amyloid burden were apparent despite improved behavioral performance [46]. There is evidence to support that insoluble Aβ has relatively low toxicity, as it is weakly correlated with synapse loss, neuronal death, and cognitive impairment [47–49]. In fact, it has been hypothesized that insoluble Aβ plaques may actually be a mechanism for soluble Aβ removal, and may serve as a sink for pathological soluble Aβ deposition to mitigate the harmful effects of the species [50–52]. One study even found that accelerating fibrilization of Aβ reduced the levels of soluble oligomers and alleviated behavioral deficits [51]. Further investigations into the mechanisms driving exercise-induced changes in insoluble Aβ pathology are necessary.

Despite increased insoluble Aβ pathology seen following high-volume exercise, CVE reduced the expression of several inflammatory cytokines, including TNF-α, IL-6, IL-1, and IFN-γ; these decreases were similar across all levels of CVE. It was previously shown that 3 weeks of wheel running alters neuroinflammatory profiles in the hippocampus of aged Tg2576 mice, associated with improved behavioral outcomes; these effects were seen in the absence of an effect on amyloid burden [15]. We previously demonstrated that treating Tg-SwDI mice with minocycline reduces inflammation and ameliorates behavioral deficits in the absence of altered amyloid pathology [30], providing further evidence that interventions targeting neuroinflammation may have therapeutic potential. Our current findings are in agreement with previous studies that have shown that exercise reduces innate immunity [53] and inflammatory cytokine expression in healthy mice and mouse models of AD associated with improved cognitive performance [44, 54, 55], as well as in aging and AD clinical populations [56–58]. It is likely that CVE reduces microglial activation (either in quantity of microglia or degree of reactivity). Previous studies have shown that microglia isolated from the brains of aged exercised mice exhibit reduced activation [59], and that exercise decreases basal levels of proliferation of microglia in aged mice [60]. Moreover, exercise has been shown to attenuate microglial sensitization, as measured by the expression of TNF-α, IL-1β, and IL-6 gene following in vitro LPS administration to hippocampal microglia from aged rats [61]. Importantly, the observed exercise-induced attenuation of neuroinflammation persisted nearly a month between the end of the intervention period and time of tissue collection, suggesting a chronic protective effect of exercise; however, the exact duration of this effect is unknown. A future cross-sectional study could address this important issue.

## Conclusions

This study represents the first assessment of the effects of life-long voluntary CVE on CAA symptomology. Additionally, we tested several access periods (“doses”) of exercise, modeled by varying length of daily access to a running wheel, resulting in exercise regimens that varied in both volume and estimates of intensity. The major finding of this study is that the expression of several inflammatory cytokines was similarly attenuated by all exercise regimens, while having no observable effect on CAA pathology. Additionally, high-volume exercise increased insoluble Aβ burden. Exercise also produced improvements in motor function, exploration, anxiety-like behavior, sociability, and short-term memory for a displaced object. No rescue of spatial learning and memory deficits characteristically displayed by Tg-SwDI mice in the Barnes maze were seen with exercise; however, these results could be confounded by exercise’s effects on motivational factors to complete the task (e.g., anxiety provoked by bright open spaces). Taken together, these findings suggest that *consistent*, *long-term* CVE can exert anti-inflammatory effects in the brain, in addition to improving behavioral outcomes related to motor, temperament, and sociability components of broad behavior deficits found in dementias. Future studies could assess whether shorter interventions may be effective, as well as the value of a low-intensity, low-volume regimen. Additionally, it is of interest to determine whether exercise interventions are purely preventative or whether they would also be effective if they begin later, at a time when more advanced pathology and severe neuroinflammation are in place.

## Data Availability

The datasets used and/or analyzed during the current study are available from the corresponding author on reasonable request.

## Abbreviations

AD: Alzheimer’s disease
ANOVA: Analysis of variance
APP: Amyloid precursor protein
Aβ: Amyloid-beta protein
BDNF: Brain-derived neurotrophic factor
CAA: Cerebral amyloid angiopathy
CVE: Cardiovascular exercise
ELISA: Enzyme-linked immunosorbent assay
IFN-ɣ: Interferon-gamma
IL-1: Interleukin-1
IL-1β: Interleukin-1β
IL-6: Interleukin-6
NOR: Novel object recognition
qPCR: Quantitative polymerase chain reaction
SEM: Standard error of the mean
TNF-α: Tumor necrosis factor alpha
WT: Wild-type
